# Flint’s Pill in Chlorosis

**Published:** 1891-08

**Authors:** 


FLINT'S PILL IN CHLOROSIS.—At University Hospital Dr. Fussell kept a record of 10 cases which had been observed for a long time. The improvement was gradual, marked, permanent, blood improving in proper ratio with other symptoms; patients becoming able to resume work after treatment which they were not able to do before. He believes that the experience with the pills in the hospital and cases reported by Dr. Flint establish that large doses of iron are not necessary in all cases of chlorosis.

Formula for Flint's pills:

R. Sodium chloride, 3 drachms
Potassium chloride, 9 grains
Potassium sulphate, 6 grains
Potassium carbonate, 3 grains
Sodium carbonate, 36 grains
Magnesium carbonate, 3 grains
Calcium phosphate, half drachm
Calcium carbonate, 3 grains
Reduced iron, 27 grains
Carbonate of iron, 3 grains.

S. In capsules No. 60. Two capsules 3 times daily at eating.—*Udiversity Med. Magazine, July, 1891.*

